# Pharmacokinetic Evaluation of a DSPE-PEG_2000_ Micellar Formulation of Ridaforolimus in Rat

**DOI:** 10.3390/pharmaceutics5010081

**Published:** 2012-12-27

**Authors:** Connie M. Remsberg, Yunqi Zhao, Jody K. Takemoto, Rebecca M. Bertram, Neal M. Davies, Marcus Laird Forrest

**Affiliations:** 1 College of Pharmacy, Department of Pharmaceutical Sciences, Washington State University, Pullman, WA 99163, USA; E-Mails: remsbergc@medsfgh.ucsf.edu (C.M.R.); jodyt@hawaii.edu (J.K.T.); rebecca.bertram@email.wsu.edu (R.M.B.); Neal.Davies@ad.umanitoba.ca (N.M.D.); 2 Department of Pharmaceutical Chemistry, University of Kansas, Lawrence, Kansas 66047, USA; E-Mail: yzhao@ku.edu (Y.Z.); 3 Faculty of Pharmacy, University of Manitoba, Winnipeg R3T 2N2, Canada

**Keywords:** ridaforolimus, preclinical pharmacokinetics, polymeric micelle, DSPE-PEG_2000_

## Abstract

The rapamycin analog, ridaforolimus, has demonstrated potent anti-proliferative effects in cancer treatment, and it currently is being evaluated in a range of clinical cancer studies. Ridaforolimus is an extremely lipophilic compound with limited aqueous solubility, which may benefit from formulation with polymeric micelles. Herein, we report the encapsulation of ridaforolimus in 1,2-distearoyl-*sn*-glycero-3-phosphoethanolamine-*N*-methoxy-poly(ethylene glycol 2000) (DSPE-PEG_2000_) via a solvent extraction technique. Micelle loading greatly improved the solubility of ridaforolimus by approximately 40 times from 200 μg/mL to 8.9 mg/mL. The diameters of the drug-loaded micelles were 33 ± 15 nm indicating they are of appropriate size to accumulate within the tumor site via the enhanced permeability and retention (EPR) effect. The DSPE-PEG_2000_ micelle formulation was dosed intravenously to rats at 10 mg/kg and compared to a control of ridaforolimus in ethanol/PEG 400. The micelle significantly increased the half-life of ridaforolimus by 170% and decreased the clearance by 58%, which is consistent with improved retention of the drug in the plasma by the micelle formulation.

## 1. Introduction

**Figure 1:**
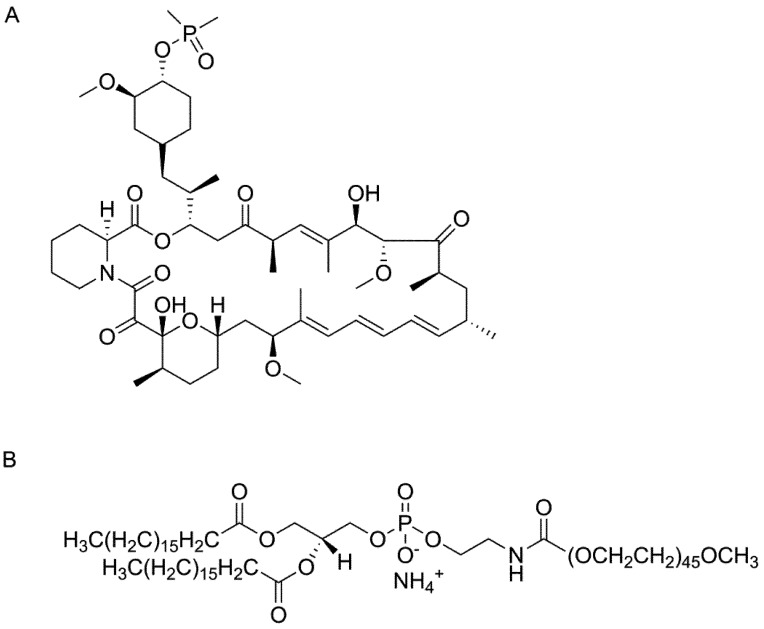
Chemical structure of (**A**) ridaforolimus and (**B**) DSPE-PEG_2000_.

Ridaforolimus, also known as deforolimus, AP-23573, and MK8669, is a non-prodrug rapamycin analog (Figure 1A). It is a large lipophilic (MW: 990 g/mol; XlogP: 5.9) carboxylic lactone-lactam macrolide antibiotic with potent anti-proliferative and immunosuppressive effects. Ridaforolimus inhibits the mammalian target of rapamycin (mTOR), a serine/threonine kinase that is expressed throughout most mammalian cells and that is involved in a variety of cell signaling cascades [1]. Inappropriate activation of mTOR has been implicated in the pathogenesis of a range of cancers and can account for tumor proliferation and growth [1]. Ridaforolimus has been shown to inhibit mTOR and has demonstrated anti-proliferative activity against several cancer types *in vitro* and *in vivo* [2].

Ridaforolimus is currently undergoing numerous clinical trials for a range of cancers both as a monotherapy and in combination with other chemotherapeutics including a Phase III clinical trial for metastatic soft-tissue and bone sarcoma. Formulations being evaluated in these clinical trials include an intravenous formulation given as an infusion and an oral formulation. The pharmacokinetic results from these studies suggest ridaforolimus follows non-linear kinetics following oral and intravenous administration as indicated by non-linear increases in area under the curve (AUC) and concentration maximum (*C*_max_) with increases in dose [3]. Changes in clearance (CL) and volume of distribution at steady state (*V*_ss_) are also reported to change with dose [3]. It has been suggested that this nonlinearity is consistent with saturation of the red blood cell compartment which contains a substantial amount of FKBP-12, the binding protein of ridaforolimus and other rapamycin analogs [4]. This strong partitioning of ridaforolimus to the red blood cell compartment may hinder the accessibility of ridaforolimus into solid tumor sites [5].

Ridaforolimus and other derivatives of rapamycin including temsirolimus and everolimus differ from rapamycin at the C-42 position. In the case of ridaforolimus, the C-42 position consists of a dimethylphosphinate substitution, which increases the aqueous solubility of ridaforolimus (*ca*. 200 μg/mL) in comparison to rapamycin (*ca.* 2.6 μg/mL) [6]. Although improved in relation to rapamycin, the solubility of ridaforolimus is still poor and has likely hindered development of clinical intravenous formulations. This cannot be verified, however, as the only formulation information published to our knowledge includes that of animal studies where ridaforolimus was solubilized in a complicated vehicle of dimethyl acetamide/polysorbate 80/polyethylene glycol-400/water (10/10/40/40, *v*/*v*/*v*/*v*) [7].

Further increasing the solubility of ridaforolimus through the use of nanocarrier technology may decrease the amount of ridaforolimus that partitions to the erythrocyte compartment and thereby allow for easier prediction of pharmacokinetic parameters. A specific class of nanocarriers that has a large solubilization capacity for poorly water soluble molecules are the pegylated phospholipid (PPL) micelles [8]. These self-assembled micelles are capable of solubilizing a wide range of hydrophobic molecules, and they are a potential tool for the safe formulation and delivery of antitumor agents to tumors without the inclusion of potentially harmful surfactants and excipients [5,8,9]. Due to their nanoscopic dimensions (typically <70 nm), PPL micelles can leave leaky vasculature, accumulating in tumors via the enhanced permeability and retention effect (EPR), where they remain due to poor lymphatic clearance [8]. An outer coat of poly(ethylene glycol) (PEG) imparts “stealth” characteristics to micelles, allowing them to circulate for long periods of time and avoid the mononuclear phagocyte system (MPS). These effects potentially reduce non-specific toxicities of the chemotherapeutic. Therefore, encapsulation of ridaforolimus in a micelle nanocarrier may limit its systemic toxicities specifically its dose-limiting toxicity of mucositis [3].

Previous investigations have confirmed that micelle formulations of poly(ethylene glycol)-block-poly(caprolactone) (PEG-b-PCL) can substantially increase the solubility and circulation time of rapamycin, while also greatly reducing the non-specific toxicity [5]. We hypothesize that encapsulation of ridaforolimus in the PPL micelle polymer 1,2-distearoyl-*sn*-glycero-3-phosphoethanolamine-*N*-methoxy-poly(ethylene glycol 2000) (DSPE-PEG_2000_) may allow for improved solubility while limiting the partition to erythrocytes. DSPE-PEG_2000_ (Figure 1B), was chosen due to its promising kinetic and thermodynamic stability and biocompatibility DSPE-PEG_2000_ has received regulatory approval in the US and/or Europe in three formulations of doxorubicin (Doxil, LipoDox and Thermodox). Herein, we report the pharmacokinetics of a DSPE-PEG_2000_ formulation of ridaforolimus in rats. To our knowledge, this is the first report of the pharmacokinetics of ridaforolimus in rats and the first attempt to solubilize ridaforolimus in any type of nanocarrier.

## 2. Results and Discussion

### 2.1. Formulation of Ridaforolimus in DSPE-PEG_2000_ Micelles

**Figure 2:**
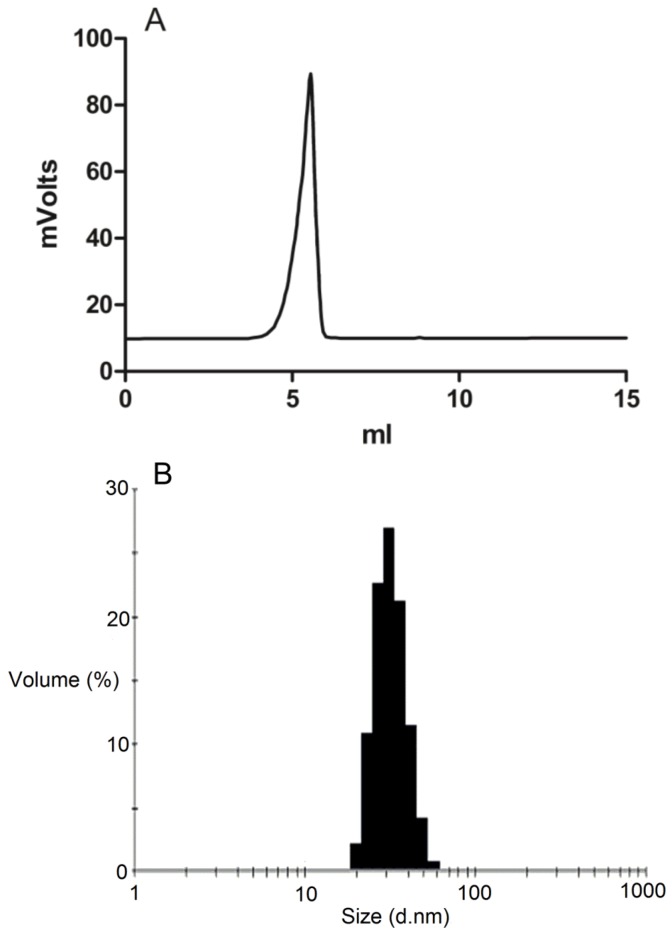
(**A**) Gel permeation chromatography (GPC) of ridaforolimus loaded DSPE-PEG_2000_ micelles in water mobile phase at 0.8 mL/min with evaporative light scattering detector (ELSD) detection; (**B**) Size distribution of ridaforolimus loaded DSPE-PEG_2000_ micelles indicating an average diameter of 33 nm.

The formation of drug loaded DSPE-PEG2000 was confirmed by gel permeation chromatography (GPC) study (Figure 2A). Loading of ridaforolimus into DSPE-PEG_2000_ micelles greatly improved the aqueous solubility of ridaforolimus by approximately 40 times from 200 μg/mL to 8.9 mg/mL [6]. The diameters of the drug-loaded micelles as determined by DLS were 33 ± 15 nm (Figure 2B). This size range has been shown appropriate for extended circulation time *in vivo* and accumulation within the tumor through the EPR effect [10].

### 2.2. Micelle Characterization

Micelles appeared as a distinct peak on SEC, with a retention time of 6.966 min for the DSPE-PEG_2000_ micelles (873,000 Da using PEG standards) (Figure 2A). The micelle fractions were collected and assayed for drug content by HPLC with UV detection. Ridaforolimus was found in the micelle GPC peak, and the HPLC retention time was identical to the standard with no additional peaks. The drug loading efficiency (DL%) for 10% (*w*/*w*) drug loaded micelles was 7.194% ± 0.143% and the encapsulation efficiency (EE%) was 77.519% ± 1.658%.

**Figure 3:**
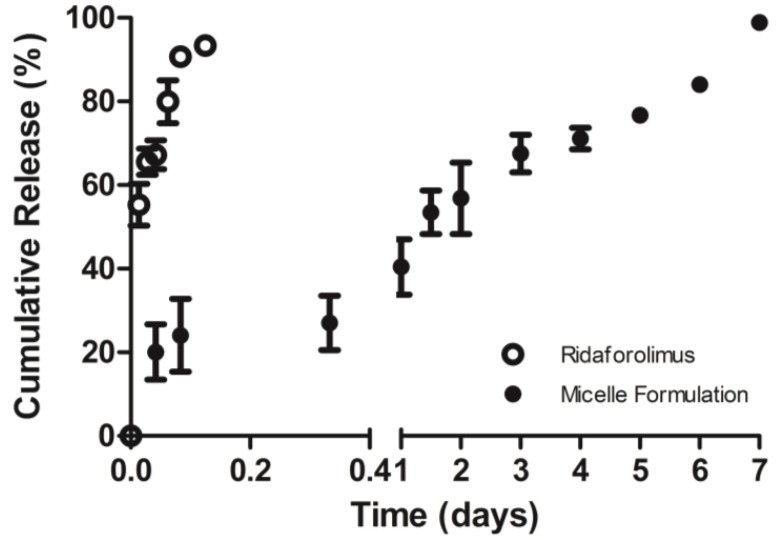
Release profile of ridaforolimus in PEG-DSPE_2000_ micelles (●) compared to free drug in PBS (○) (mean ± SD) (*n* = 3).

The *in vitro* release profiles of free drug and micelle formulation of ridaforolimus were investigated at simulated *in vivo* conditions using a PBS bath at pH 7.4 and 37 °C (Figure 3). The *in vitrot*_50%_ was improved from less than 20 min to 1.5 days and *t*_90%_ was increased from 1 h to 6.5 days (Figure 3).

### 2.3. Micelle Formulation Effects on Cellular Uptake of the Drug

**Figure 4:**
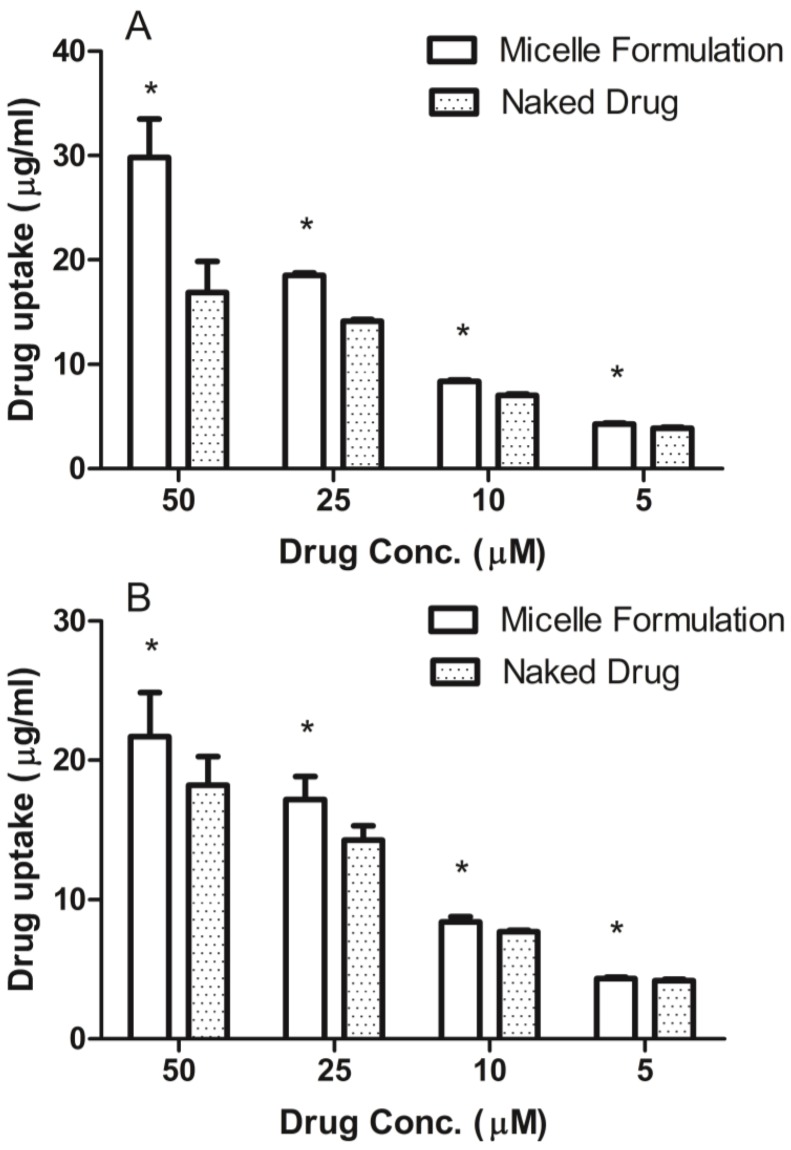
The micelle formulation improved ridaforolimus uptake by DU145 cells (**A**) and MDA-1986 cells (**B**) (mean ± SD) (* *p* < 0.01 between formulations the same concentration).

The DU145 and MDA-1986 cells were treated with naked ridaforolimus and ridaforolimus loaded DSPE-PEG_2000_ micelles. After a 6-h treatment, the drug uptaken by the cells was quantified by HPLC (Figure 4). Micelle formulation significantly improved the cellular uptake at all concentrations in both DU145 and MDA-1986 cells. In addition, DMSO was not required to solubilize ridaforolimus in the micelle formulation.

### 2.4. Pharmacokinetic Evaluation of a DSPE-PEG_2000_ Formulation of Ridaforolimus

Tolerability and the pharmacokinetic disposition of two ridaforolimus formulations were assessed. No observable differences were seen in the tolerability of the ethanol/PEG 400 formulation and DSPE-PEG_2000_ micelle formulation among blinded observers. No tolerability differences were expected as the appearance of the toxicities associated with mTOR inhibitors are most often delayed (e.g., mucositis).

**Figure 5:**
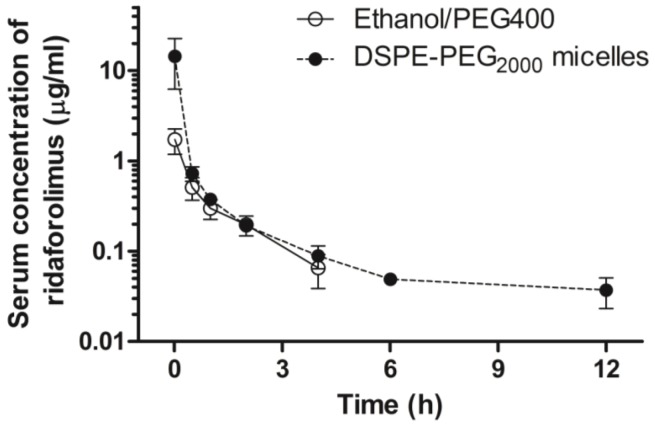
Concentration-time profile in serum of ridaforolimus after intravenous administration of control ridaforolimus formulation in ethanol/PEG 400 and ridaforolimus in DSPE-PEG_2000_ micelles. The dose for all the formulations was 10 mg/kg to rats (mean ± SEM, *n* = 7 per group).

Pharmacokinetic differences between the micellar and ethanol/PEG 400 formulations of ridaforolimus following intravenous administration are illustrated in Figure 5. Ridaforolimus in the control formulation of ethanol/PEG400 exhibited a rapid distribution and elimination from the body where concentrations fell below detectable limits after 4 h post-dose. Solubilization of ridaforolimus in DSPE-PEG_2000_ micelles provided a slightly more sustained release and quantifiable presence of ridaforolimus in serum up to 12 h. Non-compartmental analysis of the pharmacokinetic parameters of these formulations are reported in Table 1. The micelle nanocarrier formulation increased elimination half-life (*t*_1/2_) by 1.7-fold and decreased the clearance by 0.6 fold. This may indicate that the micelle formulation increases the serum circulation time of ridaforolimus *in vivo*. The micelle formulation also increased the AUC by 3.7-fold and decreased the *V*_ss_ by 1.8-fold, but these were not statistically significant (*p* < 0.05).

**Table 1:** Pharmacokinetics of ridaforolimus after IV administration of control ridaforolimus in ethanol/PEG 400 and ridaforolimus in DSPE-PEG_2000_ micelles. The intravenous dose for all the formulations was 10 mg/kg to rats (mean ± SEM, *n* = 7 per group). * Significantly different from formulation in ethanol/PEG 400 (*p* < 0.05).

PK Parameters	Ridaforolimus in ethanol/PEG 400 (mean ± SEM)	Ridaforolimus in DSPE-PEG_2000_ micelles (mean ± SEM)
AUC_0→∞_ (h × µg/mL)	1.40 ± 0.23	5.16 ± 2.13
*V*_ss_ (l/kg)	10.82 ± 2.10	6.05 ± 2.10
Cl_total_ (l/h/kg)	8.31 ± 1.58	3.45 ± 0.79 *
*t*_1/2_ (h)	1.20 ± 0.11	3.18 ± 0.64 *
MRT (h)	1.34 ± 0.15	1.57 ± 0.51

Previous attempts to solubilize highly lipophilic compounds with polymeric micelles have showed dramatic alteration in the pharmacokinetics and biodistribution of these compounds. For instance, encapsulation of paclitaxel prodrugs into PEG-b-PCL micelles increased the AUC_0__→∞_ of the prodrug 54-fold in comparison to control while improving tolerability and altering paclitaxel’s biodistribution [11]. For unclear reasons, solubilization of ridaforolimus in DSPE-PEG_2000_ micelles did not dramatically increase the residence time or the overall systemic exposure of ridaforolimus. This may be indicative of a lack of *in vivo* stability or an inability to evade the mononuclear phagocyte system. DSPE-PEG is well established to have detergent-like properties that may disrupt the balance of hydrophobicity and hydrophilicity of the nanocarrier. This is most well known in liposomes where DSPE-PEG is a common PEGylate choice yet can also be responsible for disrupting the lipid membrane [11].

No other reports of solubilization of ridaforolimus in micelles or other forms of nanocarriers are available in the biomedical literature. However, there are reports of the encapsulation of rapamycin, the parent drug of ridaforolimus, using PEG-b-PCL and DSPE-PEG_2000_ micelles [5,12,13]. The pharmacokinetics of rapamycin in these micelles has only been evaluated with the PEG-b-PCL formulation. The PEG-b-PCL greatly improved the tolerability of rapamycin with decreased distribution of rapamycin into brain tissue [5]. The formulation, however, had no significant effect of the pharmacokinetic disposition of rapamycin with only a longer *t*_1/2_ being significant [5]. These results appear to be in accordance with the results found in this study.

To our knowledge, this is the first report of the pharmacokinetics of ridaforolimus in rats. The results reported herein vary greatly from those reported in clinical Phase I trials of ridaforolimus in humans suggesting species dependent pharmacokinetics. Within these human trials, a much long half-life of 45–52 h is reported [3]. Possible differences may stem from limits reached analytically as the LC/MS method reported here could detect ridaforolimus only to 50 ng/mL while LC/MS/MS used in clinical studies allowed detection to 0.5 ng/mL [3]. This may have prevented accurate estimation of the terminal phase of elimination. Additionally, data reported within were collected from rat serum rather than whole blood. The concentrations of ridaforolimus in the serum component may have simply been too low for proper analytical quantitation.

It was hypothesized at the beginning of this study that encapsulation within DSPE-PEG_2000_ micelles would greatly alter the pharmacokinetic parameters of ridaforolimus. A decrease in volume of distribution was anticipated due to an expected decrease in partitioning of ridaforolimus to the red blood cell compartment. Limiting red blood cell partitioning could have theoretically limited saturation of the substantial amount of FKBP-12 binding protein within erythrocytes. This saturation has been suggested as a possible reason for the lack of dose proportionality with ridaforolimus and other rapamycin analogs. Taken together, it can be speculated that it may have been possible to improve the dose proportionality of ridaforolimus by solubilizing in a nanocarrier system. However, no significant changes in volume of distribution were seen, but there were trends for a decrease in volume.

These are the first of results in the literature describing the pharmacokinetics of ridaforolimus in rats as well as the first results presenting its formulation via nanocarriers. Other work in our laboratory continues to explore solubilizing lipophilic drugs in nanocarriers and the effects these formulations have on disposition of these drugs.

## 3. Experimental Section

### 3.1. Chemical and Reagents

Ridaforolimus was purchased from LC Labs (Woburn, WA, USA). 1,2-distearoyl-sn-glycero-3-phosphoethanolamine-*N*-[methoxy(polyethylene glycol)-2000] (DSPE-PEG_2000_) copolymer was purchased from Avanti Polar Lipids (Alabaster, AL, USA). HPLC-grade methanol and water were purchased from J.T. Baker (Philipsburg, NJ, USA). Other chemicals were of analytical grade. Healthy male Sprague-Dawley rats were obtained from Simonsen Labs (Gilroy, CA, USA). Ethical approval for animal experiments was obtained from Institutional Animal Care and Use Committee of Washington State University

### 3.2. Preparation of Drug Formulations

Ridaforolimus loaded micelles were prepared by solvent evaporation technique. Specifically, ridaforolimus and DSPE-PEG_2000_ (Avanti Polar Lipids, Inc.) were solubilized in 1:1 acetone and methanol. The solution was then added in a drop-wise fashion to vigorously stirred ddH_2_O using a syringe pump. The organic solvent was removed by stirring under an air purge. Solutions were then filtered through a 0.22-μm polyestersulfone filter to sterilize and remove unincorporated drug. Solutions were protected from light and stored at 4 °C until administered.

A control formulation for intravenous administration of ridaforolimus was made by dissolving ridaforolimus in ethanol followed by addition to PEG400 to form a final formulation of 1:1 ethanol:PEG. The resulting mixture was vortexed until clear and passed through a 0.22-μm polyestersulfone filter.

Formulation concentrations were determined using a Shimadzu LC-2010A (Kyoto, Japan). A Phenomenex^®^ Luna C_18_ (2) analytical column (250 × 4.6 mm; 5 μm) was used with UV detection at 290 nm (CA, USA). The mobile phase consisted of acetonitrile, water and formic acid (30:70:0.04, *v*/*v*/*v*) that was filtered and degassed prior to use with isocratic separation carried out at a flow rate of 0.3 mL/min. Hydrodynamic diameters of DSPE-PEG_2000_ micelles were performed using Dynamic Light Scattering (DLS) with a Malvern Nano ZS instrument and DTS software (Malvern Instruments Ltd., Malvern, Worcestershire, UK).

### 3.3. Micelle Characterization

The formation of DSPE-PEG_2000_ micelles was determined by gel permeation chromatography (GPC) using a Shimadzu 2010CHT system with 0.8 mL/min ddH_2_O as the mobile phase on a Shodex OHpak-803 HQ column (Showa Denko America, NY, USA) thermostated at 40 °C An evaporative light scattering detector (ELSD-LTII, Shimadzu, Lenexa, KS, USA) was used to detect the particles. Narrow molecular weight distribution polyethylene glycols (Scientific Polymer Products, Ontario, NY, USA) were used as standards for GPC analysis. To confirm the drug was encapsulated in the micelles, GPC peak fractions were collected and dried by Speed-Vac. The dried micelle fractions were redissolved in methanol and then analyzed by reversed-phase HPLC.

The drug loading efficiency (DL%) and encapsulation efficiency (EE%) of ridaforolimus in DSPE-PEG_2000_ micelles were calculated according to the following equations:

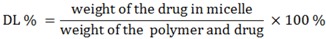
(1)

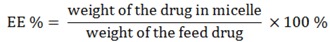
(2)


The *in vitro* release of the drug from DSPE-PEG_2000_ micelles into PBS (pH 7.4) was done by a dialysis method. Dialysis was carried out at 37 °C under sink condition using a 10-kDa MWCO dialysis tubing (SnakeSkin^®^, Thermo Scientific Inc., Rockford, IL, USA). The initial volume of drug-loaded micelles in the dialysis tubing was 2 mL and the sink solution was 4 L. The PBS was changed several times per day to maintain sink conditions. After pre-determined time intervals, samples were withdrawn from the dialysis bag and analyzed by a reversed phase HPLC column (TSK-GEL^®^ ODS-100Z, Tosoh Bioscience) at 50 °C with UV detection at 290 nm.

### 3.4. Cellular Uptake of DSPE-PEG_2000_ Micelles

Prostate cancer cell line, DU145, was maintained in RPMI-1640 medium and head and neck cancer cell line, MDA-1986, was kept in Dulbecco Modified Eagle Medium (DMEM). Both medium were supplemented with 10% fetal bovine serum (Hyclone Laboratory Inc., Logan, UT, USA). Cells were plated in 96-well flat-bottomed plates at a concentration of 10,000 cells per 90 μL of growth medium. After the cells attached to the surface, ridaforolimus dissolved in DMSO or encapsulated in DSPE-PEG_2000_ micelles was added at concentrations of 0, 5, 10, 25, 50 µM. After a 6-h treatment, the cell culture medium was removed and analyzed for extracellular drug content by HPLC.

### 3.5. LC/MS Analysis of Ridaforolimus

The LC/MS system was a Shimadzu LCMS-2010 EV liquid chromatograph mass spectrometer system (Kyoto, Japan) connected to the LC portion consisting of two LC-10AD pumps, a SIL-10AD VP auto injector, a SPD-10A VP UV detector, and a SCL-10A VP system controller was used. Data analysis was accomplished using Shimadzu LCMS Solutions Version 3 software. A Phenomenex^®^ Luna C_18_ (2) analytical column (250 × 4.6 mm; 5 µm) was used with a mobile phase of methanol, water, and formic acid (90:10:0.1, *v*/*v*/*v*) modified with 2 mM ammonium acetate at a flow rate of 0.5 mL/min. Pterostilbene was used as an internal standard. Positive selective ion monitoring (SIM) was used for detection of ridaforolimus at *m*/*z* 1012.60 (sodium adduct) and pterostilbene at *m*/*z* 257. Sodium adduct formation of other immunosuppressants have been reported [14,15]. This LC/MS method was linear over 0.05–10 μg/mL concentration range with a lower limit of quantitation (LLOQ) of 0.05 μg/mL. Interday precision and accuracy for the method were within the acceptance criteria of <15%.

### 3.6. Surgical Procedures

Male Sprague-Dawley rats (~250 g) were obtained from Simonsen Labs (Gilroy, CA, USA) and given food (Purina Rat Chow 5001) and water *ad libitum* in our animal facility for at least 3 days before use. Rats were housed in temperature-controlled rooms with a 12 h light/dark cycle. The day before the pharmacokinetic experiment, the right jugular veins of the rats were catheterized with sterile silastic cannula (Dow Corning, Midland, MI, USA) under isoflurane anesthesia. After cannulation, the Intramedic PE-50 polyethylene tubing (Becton, Dickinson and Company, Franklin Lakes, NJ, USA) connected to the cannula was exteriorized through the dorsal skin. The cannula was flushed with 0.9% saline. The animals were transferred to metabolic cages and fasted overnight. Animal use protocols were approved by The Institutional Animal Care and Use Committee at Washington State University.

### 3.7. Pharmacokinetic Study

On the days of the experiment, animals were intravenously administered a single bolus injection of either ridaforolimus in ethanol/PEG 400 (10 mg/kg, *n* = 7) or the ridaforolimus in DSPE-PEG_2000_ (10 mg/kg, *n* = 7). These dosages were based on dose varying studies previously conducted in our laboratory with the structurally similar compound, rapamycin, and are in accordance with animal studies of ridaforolimus [5,7].

After dosing, serial blood samples (~0.30 mL) were collected from the cannula at 0, 1 min, and 30 min, then 1, 2, 4, 6, 12, 24, and 48 h after intravenous administration, and the cannula flushed with 0.9% saline. After dosing and after each serial blood sampling, blinded observers were present to record any visible behavior, bleeding, or change in overall appearance of the animal as signs of acute tolerability. Each blood sample was collected and following centrifugation, the serum was collected and stored at −70 °C until analyzed. 

### 3.8. Sample Preparation

To extract ridaforolimus from serum samples, an ethyl acetate extraction was used. To 100 µL of serum sample, 10 µL of IS (100 µg/mL), 200 µL HPLC-grade water, and 1 mL of ethyl acetate were added. Samples were vortexed for 30 s, centrifuged at 5000 rpm for 5 min, and the organic phase transferred to a new sample tube and dried under a stream of nitrogen gas. Dried samples were reconstituted with 100 µL of mobile phase and 10 µL injected into the LC/MS system.

### 3.9. Pharmacokinetic Analysis

Pharmacokinetic analysis was completed using data from individual rats for which the mean and standard error of the mean (SEM) were calculated for each group except for *T*_max_ which was represented as median and range. Pharmacokinetic parameters were estimated through noncompartmental analysis by the methods of Gibaldi *et al.* [16]. The apparent terminal elimination rate constant (*k*_e_) was estimated from the slope of the log–linear phase of declining serum concentration *vs*. time plot. The half-life (*t*_1/2_) was calculated using the following equation: *t*_1/2_ = 0.693/*k*_e_. The area under the concentration time curve (AUC_0→Clast_) was calculated using the linear/logarithmic trapezoidal method. Summation of AUC_0→Clast_ and the concentration at the last measured point divided by *k*_e_ yielded AUC_0→∞_. Mean residence time (MRT) was calculated by dividing AUMC_0→∞_ by AUC_0→∞_, clearance (CL) by dividing dose by AUC_0→∞_, and volume of distribution (V_ss_) by multiplying MRT by CL.

### 3.10. Statistical Analysis

Quantification was based on calibration curves constructed using peak area ratio (PAR) of ridaforolimus to internal standard, against ridaforolimus concentrations using unweighted least squares linear regression. Pharmacokinetic analysis was performed using data from individual rats for which the mean and standard error of the mean (SEM) were calculated for each group. The data were analyzed for statistical significance in Microsoft Excel^®^ using student’s t-test with a value of *p* < 0.05 being considered statistically significant.

## 4. Conclusions

The DSPE-PEG_2000_ micellar formulation dramatically improved the solubility of ridaforolimus and provided optimum sizing of the micelle particles. Upon intravenous administration of ridaforolimus loaded DSPE-PEG_2000_ micelles to rats, alterations in the pharmacokinetics of ridaforolimus could be detected. This included a significant increase in half life and decrease in clearance, and non-significant changes in AUC and volume of distribution. These results suggest that the micelle formulation may increase the retention of drug, but the micelle formulation may have limited stability *in vivo*. Since the micelle formulation significantly enhances the drug’s aqueous solubility and eliminates harmful excipients, the toxicity profile may be improved. The toxicity and efficacy in xenografts will be reported in a future study.
